# Protein Kinase D Is Increased and Activated in Lung Epithelial Cells and Macrophages in Idiopathic Pulmonary Fibrosis

**DOI:** 10.1371/journal.pone.0101983

**Published:** 2014-07-07

**Authors:** Huachen Gan, Raymond McKenzie, Qin Hao, Steven Idell, Hua Tang

**Affiliations:** 1 Department of Cellular and Molecular Biology, The University of Texas Health Science Center at Tyler, Tyler, Texas, United States of America; 2 Texas Lung Injury Institute, The University of Texas Health Science Center at Tyler, Tyler, Texas, United States of America; Cincinnati Children's Hospital Medical Center, United States of America

## Abstract

Idiopathic pulmonary fibrosis (IPF) is a relentlessly progressive and usually fatal lung disease of unknown etiology for which no effective treatments currently exist. Hence, there is a profound need for the identification of novel drugable targets to develop more specific and efficacious therapeutic intervention in IPF. In this study, we performed immunohistochemical analyses to assess the cell type-specific expression and activation of protein kinase D (PKD) family kinases in normal and IPF lung tissue sections. We also analyzed PKD activation and function in human lung epithelial cells. We found that PKD family kinases (PKD1, PKD2 and PKD3) were increased and activated in the hyperplastic and regenerative alveolar epithelial cells lining remodeled fibrotic alveolar septa and/or fibroblast foci in IPF lungs compared with normal controls. We also found that PKD family kinases were increased and activated in alveolar macrophages, bronchiolar epithelium, and honeycomb cysts in IPF lungs. Interestingly, PKD1 was highly expressed and activated in the cilia of IPF bronchiolar epithelial cells, while PKD2 and PKD3 were expressed in the cell cytoplasm and nuclei. In contrast, PKD family kinases were not apparently increased and activated in IPF fibroblasts or myofibroblasts. We lastly found that PKD was predominantly activated by poly-L-arginine, lysophosphatidic acid and thrombin in human lung epithelial cells and that PKD promoted epithelial barrier dysfunction. These findings suggest that PKD may participate in the pathogenesis of IPF and may be a novel target for therapeutic intervention in this disease.

## Introduction

Idiopathic pulmonary fibrosis (IPF), the most common form of the idiopathic interstitial pneumonias, is a chronic, relentlessly progressive and usually fatal lung disease of unknown etiology for which no effective pharmacologic treatments currently exist [1], [2], [3], [4]. IPF often demonstrates a usual interstitial pneumonia (UIP) pattern by histology and is characterized by lung epithelial cell dysfunction, lung fibroblast activation and proliferation, excessive collagen deposition, and subsequent destruction of the normal lung architecture with loss of alveolar spaces [4]. Long-term survival of IPF patients is poor, with a 5-year survival rate of only 20%. IPF is therefore more lethal than many cancers. A number of recent clinical trials of novel drugs, including interferon-γ, endothelin antagonists, the platelet-derived growth factor receptor inhibitor imatinib, tumor necrosis factor-α antibody etanercept, and anticoagulants (warfarin and heparin), have all failed to show significant benefit for IPF patients who have mild to moderate lung functional impairment. Most of these drugs showed early promise in the bleomycin-induced murine lung fibrosis model [1], [2], [3], [4], in which pulmonary fibrosis is spontaneously reversible [5]. Human IPF alternatively displays a progressive and lethal course of disease that is believed to be mediated in part by aberrant activation of lung epithelial cells [2], [4]. Hence, there is a profound unmet need for identification of novel biomarkers and key molecules or pathways that control abnormal responses of the epithelium in the pathogenesis of IPF.

The serine/threonine protein kinase D (PKD) family kinases include PKD1 (also called protein kinase Cμ-PKCμ), PKD2 and PKD3 (PKCν) [6]. PKD contains a tandem repeat of zinc finger-like cysteine-rich motifs at its N terminus that display high affinity for diacylglycerol or phorbol ester, a pleckstrin homology domain, and a C-terminal catalytic domain that shares homology with the calmodulin-dependent kinases [6]. In response to various stimuli, PKD translocates from the cytosol to different cellular compartments including the Golgi complex, nucleus and plasmas membrane to exert functions. PKD has been implicated in cell proliferation, vesicle fission and trafficking, gene expression, and rearrangement of actin cytoskeleton [6], [7]. Although PKD family kinases exhibit a homologous catalytic domain, they vary with respect to their subcellular localization, expression, and regulation [6], [7], [8]. PKD1 contains a high frequency of apolar amino acids, mainly alanine and proline at the N terminus. PKD2 has unique N- and C-terminal domains that determine its nucleocytoplasmic shuttling, activation and substrate targeting, whereas PKD3 lacks the alanine- and proline-rich regions at the N terminus and an autophosphorylation site at the C terminus [9], [10], [11]. These findings suggest functional differences among PKD isoforms. We have shown that PKD1 regulates the production of proinflammatory cytokines by vascular endothelial growth factor in endothelial cells [12] and that PKD2 is pivotal for angiogenesis [13]. We also found that both PKD2 and PKD3 were novel growth regulators in triple-negative breast cancer cells [14]. Moreover, it has been shown that PKD1 is a key modulator of macrophage activation by toll-like receptors (TLRs) [15] and that PKD inhibition suppresses microbial Ag-induced hypersensitivity pneumonitis in mice [16]. However, little is known about the regulation and functions of PKD in the context of lung epithelial cells in IPF.

To determine whether PKD is involved in the pathogenesis of IPF, we compared the cell type-specific expression and activation of PKD isoforms in IPF lung tissues with normal controls and found that PKD family kinases were increased and activated in bronchiolar and alveolar epithelial cells as well as macrophages in IPF. We further found that PKD was predominantly activated by poly-L-arginine, lysophosphatidic acid (LPA), and thrombin in human lung epithelial cells and that PKD promoted epithelial barrier dysfunction.

## Materials and Methods

### Ethics Statement

This study involved the use of de-identified lung tissue section slides from control normal subjects and IPF patients. Paraffin-embedded IPF lung tissue slides were kindly provided by the Lung Tissue Research Consortium and the paraffin-embedded normal lung tissue slides were obtained commercially. The present study was reviewed and approved by the Institutional Review Board of the University of Texas Health Science Center at Tyler.

### Antibodies and Reagents

Antibodies specifically against PKCμ/PKD1 (A-20) and vinculin were from Santa Cruz Biotechnology (Santa Cruz, CA). PKD2 antibody was from Millipore (Billerica, MA). PKD3 antibody was from Bethyl Laboratories (Montgomery, TX). Phospho-PKD (Ser744/748) antibodies and reagents for chemiluminescence detection were from Cell Signaling (Beverly, MA). Vectastain Elite ABC kit, Vector NovaRED substrate, and Vector hematoxylin QS nuclear counterstain were from Vector Laboratories (Burlingame, CA). Recombinant human transforming growth factorβ (TGFβ), epidermal growth factor (EGF), tumor necrosis factorα (TNFα), platelet-derived growth factorβ (PDGFβ), and fibroblast growth factor (FGF) were from R & D Systems (Minneapolis, MN). Thrombin, poly-L-arginine and lipopolysaccharides (*Escherichia coli* 0111:B4) were from Sigma (St Louis, MO). LPA was from Avanti (Alabaster, AL). TLR ligands Type B CpG oligonucleotide (ODN 2006), polyinosinic-polycytidylic acid (poly (I:C)), peptidoglycan (PGN), and Pam3CSK4 were from InvivoGen (San Diego, CA).

### Lung Tissue Sections

De-identified formalin-fixed paraffin-embedded lung tissue section slides from 12 patients with a diagnosis of IPF/UIP were obtained from the Lung Tissue Research Consortium which is supported by the National Heart, Lung, and Blood Institute. The IPF patients included 7 males and 5 females with a mean age of 54.2 years (range from 40 to 66 years). Formalin-fixed paraffin-embedded normal lung tissue slides from 7 subjects ranged in age from 24 to 80 years were obtained from BioChain (Newark, CA), US Biomax (Rockville, MD), IHCWorld (Woodstock, MD), ProSci (Poway, CA), and Alpha Diagnostic (San Antonio, TX), respectively.

### Immunohistochemical Analysis

Lung section slides were deparaffinized by incubation at 56°C for 30 min and subsequent xylene washes, then rehydrated by using a graded ethanol series. For antigen retrieval, the sections were incubated at 95°C for 8 min in 10 mM sodium citrate buffer (pH 6.0), then soaked in the buffer for another 30 min at room temperature. The slides were then treated with 3% H_2_O_2_ for 5 min and washed in phosphate-buffered saline with 0.1% tween-20. Immunohistochemical staining was performed using a Vectastain Elite ABC kits according to the manufacturer's instructions. Primary antibodies were diluted and applied to sections and incubated for 40 min. The dilutions of rabbit polyclonal primary antibodies were: PKD1 (A-20), 1∶500; PKD2, 1∶300; PKD3, 1∶500; and phospho-PKD (Ser744/748), 1∶50. Non-immune normal rabbit immunoglobulin served as a negative control. After incubation with biotinylated secondary antibody and ABC reagents, all the sections were incubated with peroxidase substrate Vector NovaRed for an equal amount of time to allow for suitable staining. The slides were then lightly conterstained with Hematoxylin QS, mounted with VectaMount mounting medium, examined and photographed using an Olympus BX41 microscope equipped with an Olympus DP25 digital camera. Adobe Photoshop 7.0 software was used for image processing. The staining was evaluated according to the number and intensity of immunopositive cells and scored semiquantitatively using a scale from − to + + +: negative (−), weak (+), moderate (++), or strong (+++) in different types of pulmonary cells as described previously [17].

### Cell Culture

16HBE14o- human bronchial epithelial cells [18] were kindly provided by Dr. Dieter Gruenert (University of California at San Francisco) and cultured in Dulbecco's Modified Eagle Medium (DMEM) supplemented with 10% fetal bovine serum (FBS). Primary human small airway epithelial cells and human pulmonary artery endothelial cells (HPAECs) were obtained from Lonza (Walkersville, MD), cultured in small airway growth medium or endothelial cell growth medium-2 (EGM-2) respectively, and used for experiments within 3 passages. A549 human alveolar adenocarcinoma cells were obtained from American Type Culture Collection (Manassas, VA) and cultured in RPMI-1640 medium containing 10% FBS. Primary IPF fibroblasts were isolated from the lung biopsies of IPF patients. Fibroblasts isolated from histologically normal lung tissue obtained at the time of pulmonary resection of tumors or other lesions served as control normal lung fibroblasts. These fibroblasts and their clinical origins have been characterized [19], [20] and were supplied by Dr. Cory Hogaboam, University of Michigan Medical School. The primary fibroblasts were de-identified, were cultured in DMEM supplemented with 10% FBS and were used for experiments at passages 6 to 10. In some experiments, 16HBE14o- cells were co-cultured with IPF fibroblasts or HPAECs by culturing IPF fibroblasts or HPAECs in the lower chamber below 16HBE14o- cell monolayers on permeable Transwell inserts (Corning).

### Western Blot Analysis

Western blot analysis was performed essentially as we described previously [21]. Cells were washed twice with ice-cold phosphate-buffered saline and then lysed on ice in Nonidet P-40 lysis buffer (25 mM Tris-HCl, pH 7.5, 1% Nonidet P-40, 150 mM NaCl, 10 mM NaF, 1 mM Na_3_VO_4_, 1 mM phenylmethylsulfonyl fluoride, 10 µg/ml each of leupeptin and aprotinin). Whole cell lysates at equal protein amounts were subjected to SDS-PAGE and transferred to polyvinylidene difluoride membrane. The membrane was probed with various primary antibodies as indicated and detected using the ECL system with horseradish peroxidase-conjugated secondary antibodies according to the manufacturer's protocol (Cell Signaling).

### Transepithelial Electrical Resistance (TEER)

TEER was measured with an EVOMX voltohmmeter (World Precision Instruments). The data, which subtract the basal resistance of cell-free collagen-coated Transwell inserts from each experimental point, were presented either as absolute values (Ohm×cm^2^) or changes relative to the control group as we described recently [22].

## Results

### PKD family kinases are increased in bronchiolar and alveolar epithelia as well as macrophages in IPF

**Figure 1:**
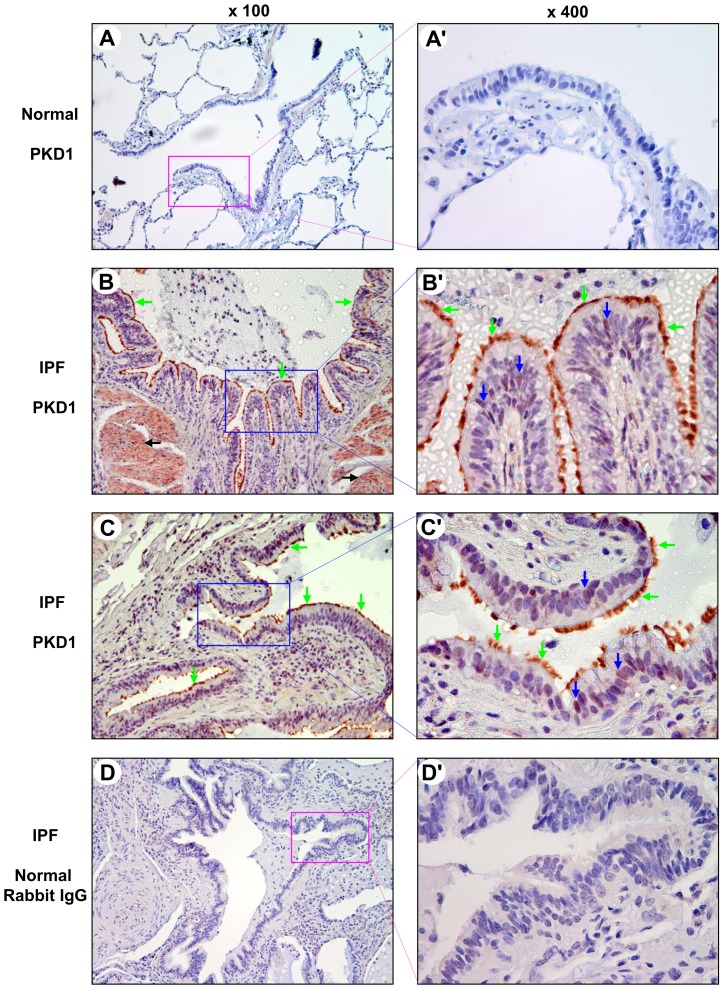
Expression of PKD1 in normal and IPF bronchiolar epithelia. Normal and IPF lung sections were subjected to immunohistochemical analysis by a PKD1 specific polyclonal antibody PKD1 (A-20) or a normal rabbit IgG at a dilution of 1∶500. A and A′: normal lung bronchiolar epithelium was negative for PKD1. The region indicated in panel A (magnification, ×100) is shown at higher magnification in A′ (×400). B–C′: in IPF lungs, PKD1 (red) was expressed abundantly in cilia (green arrows) and moderately in nuclei (blue arrows) of IPF BECs and in smooth muscle cells (B, black arrows). The regions indicated in panels B and C (×100) are shown at higher magnification in B′ and C′ (×400). D and D′: no specific signal was observed in IPF lung bronchiolar epithelium by a normal rabbit IgG. The region indicated in panel D (×100) is shown at higher magnification in D′ (×400).

We performed immunohistochemical analysis to determine the expression levels of PKD family kinases in normal and IPF lung tissue sections by using PKD isoform-specific antibodies. We used a PKD1 specific antibody PKD1 (A-20) at a dilution of 1∶500 and found that PKD1 was undetectable in bronchiolar epithelium of all the normal lungs examined (n = 7; Fig. 1, A and A′). Interestingly, we found that PKD1 was expressed abundantly in cilia and weakly to moderately in nuclei of all the IPF bronchiolar epithelial cells (BECs) (Fig. 1, B–C′; Table 1). The cilia of BECs were confirmed by an acetyl-α-tubulin (Lys40) antibody (data not shown). This observation is of particular interest as increasing evidence indicates that the disorders of small airways are involved in the pathogenesis of IPF [23], [24], [25]. Moreover, smooth muscle cells surrounding small airways from 67% (8 of 12) IPF lungs stained positive for PKD1 (Fig. 1B and Table 1). No specific signal was observed when a normal rabbit IgG was used for staining (Fig. 1, D and D′).

**Table 1:** Summary of PKD expression and activation in normal and IPF lung sections.

	1∶500 (PKD1 A-20)				1∶300 (Upstate)				1∶500 (Bethyl)				1∶50 Cell Signal)			
	PKD1				PKD2				PKD3				PKD-pS744/748			
	—	+	++	+++	—	+	++	+++	—	+	++	+++	—	+	++	+++
	Case number (%)				Case number (%)				Case number (%)				Case number (%)			
Normal Lung:																
Bronchiloar-																
SMC	7 (100)				7 (100)				7 (100)				7 (100)			
Epi.	7 (100)				7 (100)				5 (71)	2 (29)			7 (100)			
Pneumocytes	7 (100)				7 (100)				5 (71)	2 (29)			7 (100)			
Macrophages	7 (100)				6 (86)	1 (14)			6 (86)	1 (14)			6 (86)	1 (14)		
IPF Lung:																
Bronchiloar-SMC	4 (33)	5 (42)	2 (17)	1 (8)	3 (25)	5 (42)	3 (25)	1 (8)	3 (30)	4 (40)	2 (20)	1 (10)	4 (34)	6 (50)	1 (8)	1 (8)
Bronchiloar Epi.-																
Cilia		2 (17)	6 (50)	4 (33)	12(100)				10(100)					4 (33)	5 (42)	3 (25)
Nuclear		10 (83)	2 (17)		7 (58)	5 (42)				5 (50)	5 (50)		2 (17)	8 (66)	2 (17)	
Cytosol	12(100)					7 (58)	4 (34)	1 (8)		5 (50)	4 (40)	1 (10)		7 (58)	4 (34)	1 (8)
Regenerative Epi.	3 (25)	4 (33)	3 (25)	2 (17)		4 (34)	7 (58)	1 (8)	2 (20)	4 (40)	3 (30)	1 (10)	2 (17)	4 (33)	5 (42)	1 (8)
Macrophages	2 (17)	4 (33)	3 (25)	3 (25)		2 (17)	7 (58)	3 (25)		4 (40)	4 (40)	2 (20)		4 (33)	5 (42)	3 (25)
Fibroblasts	6 (50)	6 (50)			9 (75)	3 (25)			8 (80)	2 (20)			8 (67)	4 (33)		

SMC: smooth muscle cells, Epi: epithelial cells.

**Figure 2:**
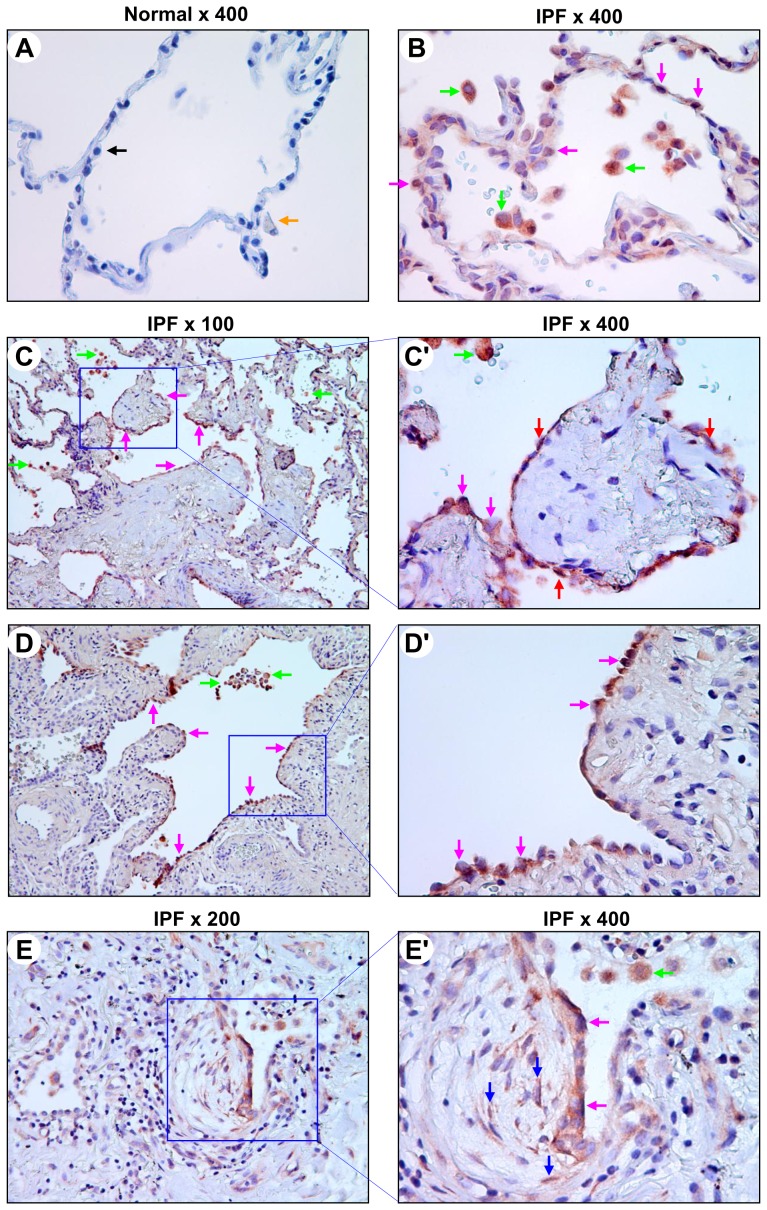
Expression of PKD1 in normal and IPF alveoli. Normal and IPF lung sections were subjected to immunohistochemical analysis by a PKD1 specific antibody PKD1 (A-20) at a dilution of 1∶500. A: normal lung AECs (black arrow) and macrophages (orange arrow) were negative for PKD1. Final magnification: ×400. B: in non-fibrotic areas of IPF lung alveoli, PKD1 (red) were expressed in the cytoplasm and nuclei of macrophages (green arrows) and AECs, including type II pneumocytes (pink arrows). Final magnification: ×400. C–D′: in the fibrotic areas of IPF lung, PKD1 (red) was expressed in flat (red arrows) and cuboidal (pink arrows) regenerative AECs lining remodeled fibrotic alveolar septa and/or fibroblast foci. Strong PKD1 immunoreactivity was also observed in macrophages (green arrows). The regions indicated in panels C and D (×100) are shown at higher magnification in C′ and D′ (×400). E and E′: PKD1 (red) were expressed in some fibroblasts or myofibroblasts (blue arrows) in fibroblasts foci of IPF lung and in regenerative AECs (pink arrows) covering the fibroblast foci as well as in macrophages (green arrow). The region indicated in panel E (×200) is shown at higher magnification in D′ (×400).

The main histopathological feature of IPF/UIP is a temporally heterogeneous appearance of the fibrotic areas with alternating areas of less affected or normal parenchyma, scattered fibroblastic foci, and honeycombing [4], [26]. In non-fibrotic areas of IPF lung alveoli, we found that PKD1 was expressed in the cytoplasm and nuclei of macrophages and alveolar epithelial cells (AECs), including type II pneumocytes (Fig. 2B). In contrast, PKD1 was not detected in any of the normal lung AECs and macrophages examined by the specific PKD1 antibody (Fig. 2A). In the fibrotic areas of IPF lung, regenerative epithelium [17], [27], [28] that participates in fibrosis remodeling of the lung was seen covering fibroblast foci and remodeled alveolar walls. As shown in Fig. 2 (C–E′) and Table 1, we found that PKD1 was expressed with a weak to strong intensity in regenerative AECs lining remodeled fibrotic alveolar septa and fibroblast foci in 75% (9 of 12) of IPF subjects examined. Strong PKD1 immunoreactivity was also observed in macrophages but not in other infiltrating inflammatory cells in the fibrotic areas. In addition, some fibroblasts or myofibroblasts in fibroblast foci showed a weak positive staining for PKD1 in 50% of the examined IPF lungs (Fig. 2, E and E′). The results of PKD1 immunohistochemical analysis are summarized in Table 1.

**Figure 3:**
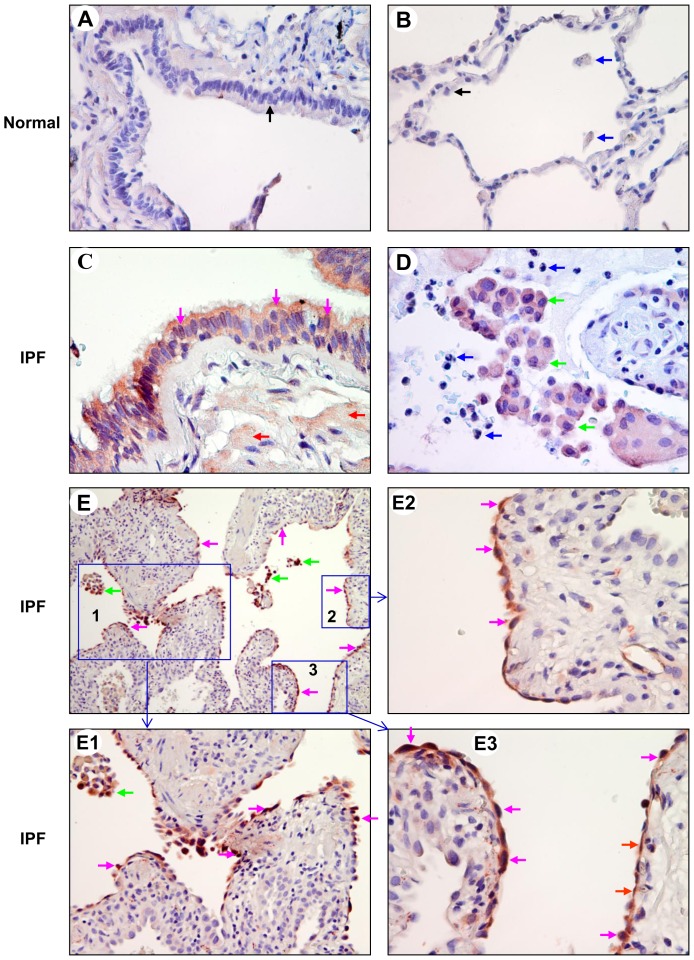
Expression of PKD2 in normal and IPF lung tissues. Normal and IPF lung sections were subjected to immunohistochemical analysis by a PKD2 specific antibody at a dilution of 1∶300. A and B: normal lung BECs and AECs (black arrow) as well as macrophages (blue arrows) were stained negative for the PKD2 antibody. Final magnification: ×400. C: in IPF bronchiolar epithelium, PKD2 (red) was expressed in the cytoplasm and nuclei of BECs (pink arrows) and in smooth muscle cells (red arrows). Final magnification: ×400. D: PKD2 (red) was expressed in macrophages (green arrows) but not neutrophils (blue arrows) in IPF lung alveoli. Final magnification: ×400. E–E3: in the fibrotic areas of IPF lung, PKD2 (red) was expressed in the flat (red arrows) and cuboidal (pink arrows) regenerative AECs lining remodeled fibrotic alveolar septa and/or fibroblast foci. It should be noted that alveolar walls grew and expanded towards the regenerative AECs overexpressing PKD2. Strong PKD2 immunoreactivity was also observed in macrophages (green arrows). The regions indicated in panel E (×100) are shown at higher magnification in E1 (×200), E2 (×400), and E3 (×400).

We next assessed the expression level of PKD2 in normal and IPF lung tissue sections by using a PKD2 specific antibody [13]. We found that PKD2 was barely detectable in normal lung BECs, AECs, and macrophages (Fig. 3, A and B). In contrast, PKD2 was expressed with a weak to strong intensity in all of the IPF lung BECs, AECs, and the infiltrated macrophages (Fig. 3, C–E3; Table 1). In particular, PKD2 was detected mainly in the cytoplasm of IPF BECs and in some cases in cell nuclei (Fig. 3C). Macrophages but not neutrophils were stained positive for PKD2 in the cytoplasm (Fig. 3D). Similar to PKD1, PKD2 was highly expressed in the flat and cuboidal regenerative AECs lining remodeled fibrotic alveolar septa and fibroblast foci in all of the IPF subjects examined (12 of 12) (Fig. 3, E–E3). It should be noted that the alveolar walls grew and expanded towards the regenerative AECs overexpressing PKD2 (Fig. 3E). In addition, PKD2 immunoreactivity was also observed in smooth muscle cells surrounding small airways (Fig. 3C) and in some fibroblasts or myofibroblasts in fibroblasts foci (Table 1).

**Figure 4:**
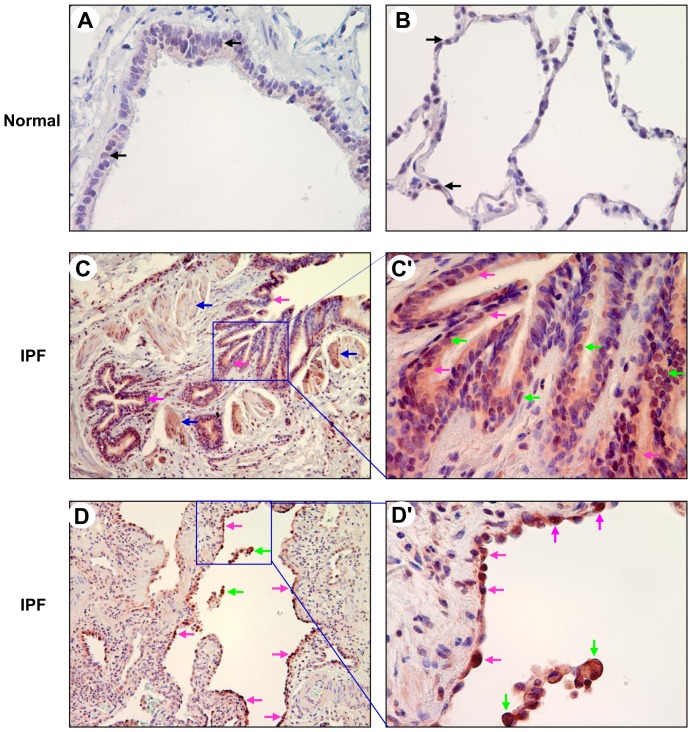
Expression of PKD3 in normal and IPF lung tissues. Normal and IPF lung sections were subjected to immunohistochemical analysis by a PKD3 specific antibody at a dilution of 1∶500. A and B: normal lung BECs and AECs (black arrows) were weakly stained in the nuclei by the PKD3 antibody. Final magnification: ×400. C and C′: in IPF bronchi, PKD3 (red) was expressed in the cytoplasm (pink arrows) and nuclei (green arrows) of BECs and in smooth muscle cells (C, blue arrows). The region indicated in panel C (×100) is shown at higher magnification in C′ (×400). D and D′: in the fibrotic areas of IPF lung, PKD3 (red) was expressed in the cuboidal regenerative AECs (pink arrows) lining remodeled fibrotic alveolar septa and/or fibroblast foci and in macrophages (green arrows). The region indicated in panel D (×100) is shown at higher magnification in D′ (×400).

We lastly examined PKD3 expression level in normal and IPF lung tissue sections by using a PKD3 specific antibody. Generally PKD3 was barely detectable in normal lung bronchiolar and alveolar epithelia as well as macrophages. In two cases (29%), PKD3 immunoreactivity could be slightly detected in the nuclei of normal lung BECs and AECs (Fig. 4, A and B). Like PKD1 and PKD2, we found that PKD3 was expressed in IPF lung BECs, regenerative AECs, and infiltrating macrophages (Fig. 4, C–D′), as summarized in Table 1. Additionally, PKD3 was also expressed in smooth muscle cells surrounding small airways (Fig. 4C). Fibroblasts or myofibroblasts in fibroblast foci stained largely negative for PKD3 (Table 1). Taken together, these findings indicate that the expression of PKD family kinases is increased in bronchiolar and alveolar epithelia as well as macrophages in IPF lungs.

### PKD family kinases are activated in bronchiolar and alveolar epithelia as well as macrophages in IPF

**Figure 5:**
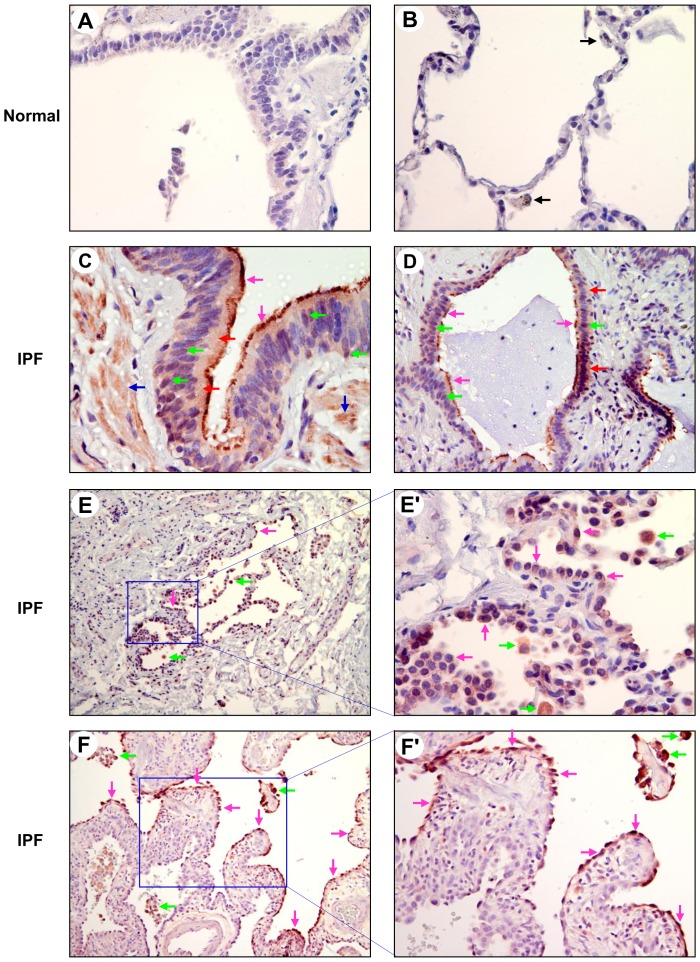
PKD family kinases are activated in bronchiolar and alveolar epithelia as well as macrophages in IPF. Normal and IPF lung sections were subjected to immunohistochemical analysis by using a phospho-specific PKD-pSer744/748 antibody at a dilution of 1∶50. A and B: normal lung BECs, AECs, and macrophages (black arrows) were all stained negative by PKD-pSer744/748 antibody. Final magnification: ×400. C and D: positive immunoreactivities (red) were detected in the cilia (pink arrows), cytoplasm (red arrows) and nuclei (green arrows) of IPF bronchiolar (C, ×400) and honeycomb cyst (D, ×200) epithelia. Positive staining was also observed in the smooth muscle cells (C, blue arrows) surrounding small airways. E–F′: in IPF alveoli, PKD phosphorylation on Ser-744/748 (red) was readily detected in most of the hyperplastic type II pneumoctyes (E and E′, pink arrows) and the regenerative AECs (F and F′, pink arrows) lining remodeled fibrotic alveolar septa and/or fibroblast foci. It should be noted that alveolar walls grew and expanded towards the regenerative AECs with activated PKDs (F and F′). Strong positive immunoractivities (red) were also observed in alveolar macrophages (green arrows). The regions indicated in panels E and F (×100) are shown at higher magnification in E′ (×400) and F′ (×200).

PKD is normally activated by a novel PKC-mediated phosphorylation of the activation loop two key serine residues [6] and thus its activation can be monitored by PKD-pSer744/748 antibody that recognizes two key phosphorylated serines at the equivalent sites among PKD isoforms. We determined the activation status of PKD family kinases in normal and IPF lung tissue sections by using the phospho-specific PKD-pSer744/748 antibody. We found that the phosphorylation of PKD on Ser-744/748 was barely detectable in normal lung BECs, AECs and macrophages (Fig. 5, A and B). In contrast, the immunoreactivities were detected with a weak to strong intensity in IPF BECs, AECs, and infiltrated macrophages by the PKD-pSer744/748 antibody (Fig. 5, C–F′; Table 1). Specifically, the cilia, cytoplasm and nuclei of IPF bronchiolar and honeycomb cyst epithelia were all stained positively by the antibody (Fig. 5, C and D). Honeycomb cysts are defined as mucus-containing cysts that contain less invaginated airway epithelium [25]. Positive staining was also observed in the smooth muscle cells surrounding small airways from 67% (8 of 12) IPF subjects (Fig. 5C and Table 1). At the alveolar level of the IPF lung tissues, Fig. 5 (E and E′) shows an area of alveolar septa surrounded by acellular collagen bundles. The alveolar walls were thickened by collagen deposition and there was a hyperplasia of type II pneumocytes, and most of the hyperplastic type II pneumoctyes were stained positive in cytoplasm and nuclei for the PKD-pSer744/748 antibody. Moreover, PKD phosphorylation on Ser-744/748 was also readily detected in the regenerative AECs lining remodeled fibrotic alveolar septa and/or fibroblast foci in 83% (10 of 12) of IPF subjects; and the alveolar walls grew and expanded towards the regenerative AECs with activated PKDs (Fig. 5, F and F′; Table 1). Moreover, we observed that macrophages in alveoli of IPF lungs were all stained positive for the PKD-pSer744/748 antibody (Fig. 5, E–F′). Additionally, fibroblasts or myofibroblasts in fibroblastic foci were variably stained positively by the PKD-pSer744/748 antibody (Table 1). These results indicate that PKD family kinases are activated in IPF bronchiolar and alveolar epithelia as well as macrophages.

### Agonist-induced activation of PKD in lung epithelial cells

**Figure 6:**
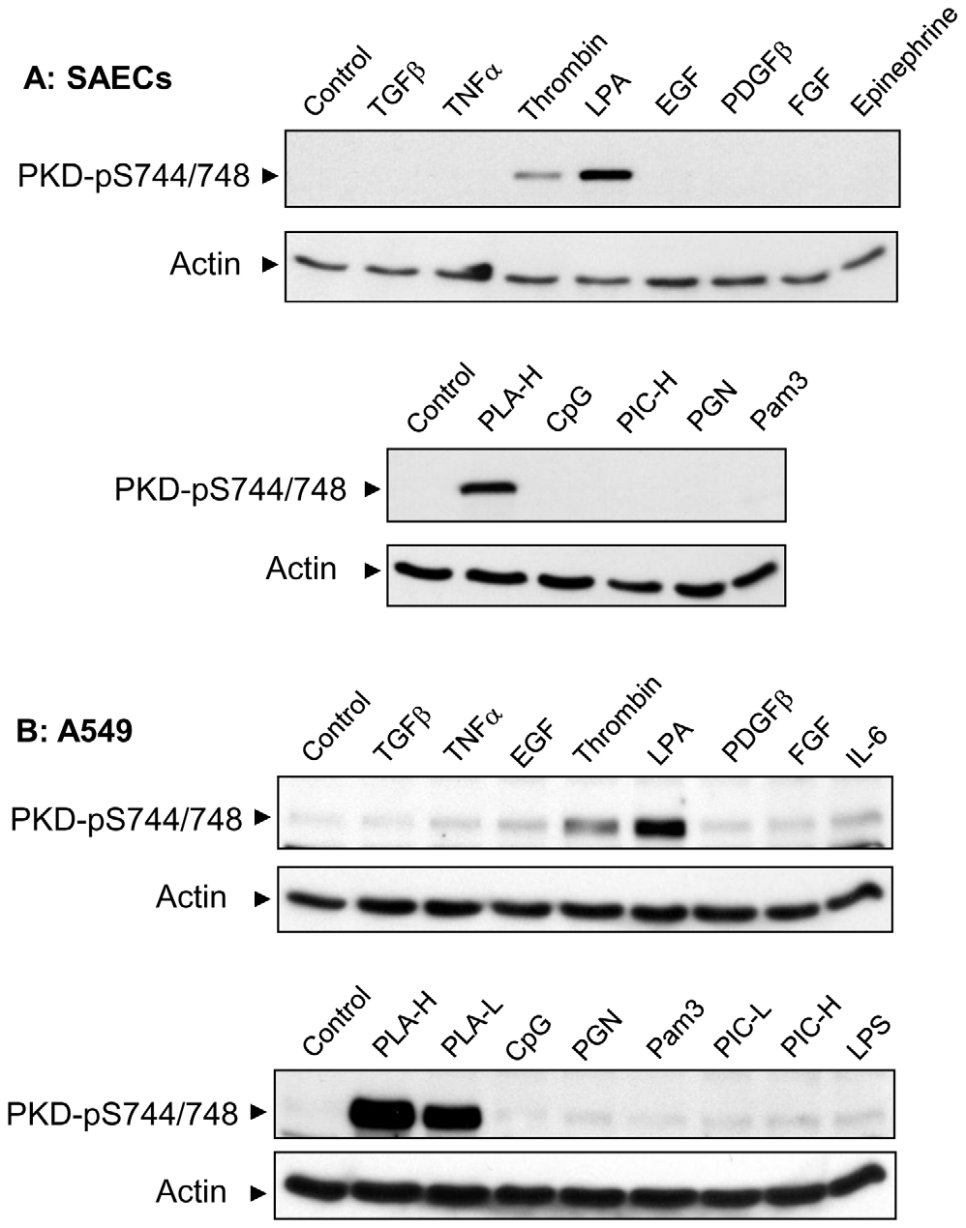
Agonist-induced activation of PKD in lung epithelial cells. Primary human small airway epithelial cells (SAECs) (A) and A549 (B) cells were starved 2 h and then treated for 10 min with control vehicle, 10 ng/ml TGFβ, 5 ng/ml TNFα, 2 U/ml thrombin, 10 µM LPA, 50 ng/ml EGF, 50 ng/ml PDFDβ, 50 ng/ml FGF, 10 µM epinephrine, 25 ng/ml interlukin-6 (IL-6), 1 µM poly-L-arginine (high molecular weight: PLA-H or low molecular weight: PLA-L), 5 µg/ml CpG oligonucleotide, 10 µg/ml poly (I:C) (high molecular weight: PIC-H or low molecular weight: PIC-L), 10 µg/ml PGN, 10 µg/ml Pam3CSK4, or 1 µg/ml lipopolysaccharides (LPS). Cell lysates at equal protein amounts (45 µg) were analyzed by Western blotting with PKD-pSer744/748 or actin antibodies as indicated. Results represent western blots of three independent experiments.

To identify agonists that activate PKD in lung epithelial cells, we next treated primary human small airway epithelial cells and A549 alveolar cell line with various receptor ligands or stimuli and performed Western blotting analysis to assess PKD activation by using the phospho-specific PKD-pSer744/748 antibody. Interestingly, we found that PKD was predominantly activated by poly-L-arginine, LPA, and thrombin through a strong phosphorylation of PKD on Ser-744/748 in both primary airway epithelial and A549 alveolar cells (Fig. 6). LPA and thrombin are profibrotic factors and have been shown to play important roles in the pathogenesis of pulmonary fibrosis [29], [30]. Poly-L-arginine is a highly charged cationic polypeptide that is similar in structure and function to the active moiety of major basic protein secreted from eosinophils [31]. In contrast, the phosphorylation of PKD on Ser-744/748 was only slightly increased by TNFα, EGF, FGF, interlukin-6, as well as TLR ligands (PGN, Pam3, Poly (I:C) and LPS) in A549 cells but not in primary airway epithelial cells (Fig. 6).

### PKD promotes lung epithelial barrier dysfunction and permeability in the presence or absence of co-cultured primary lung fibroblasts or endothelial cells

**Figure 7:**
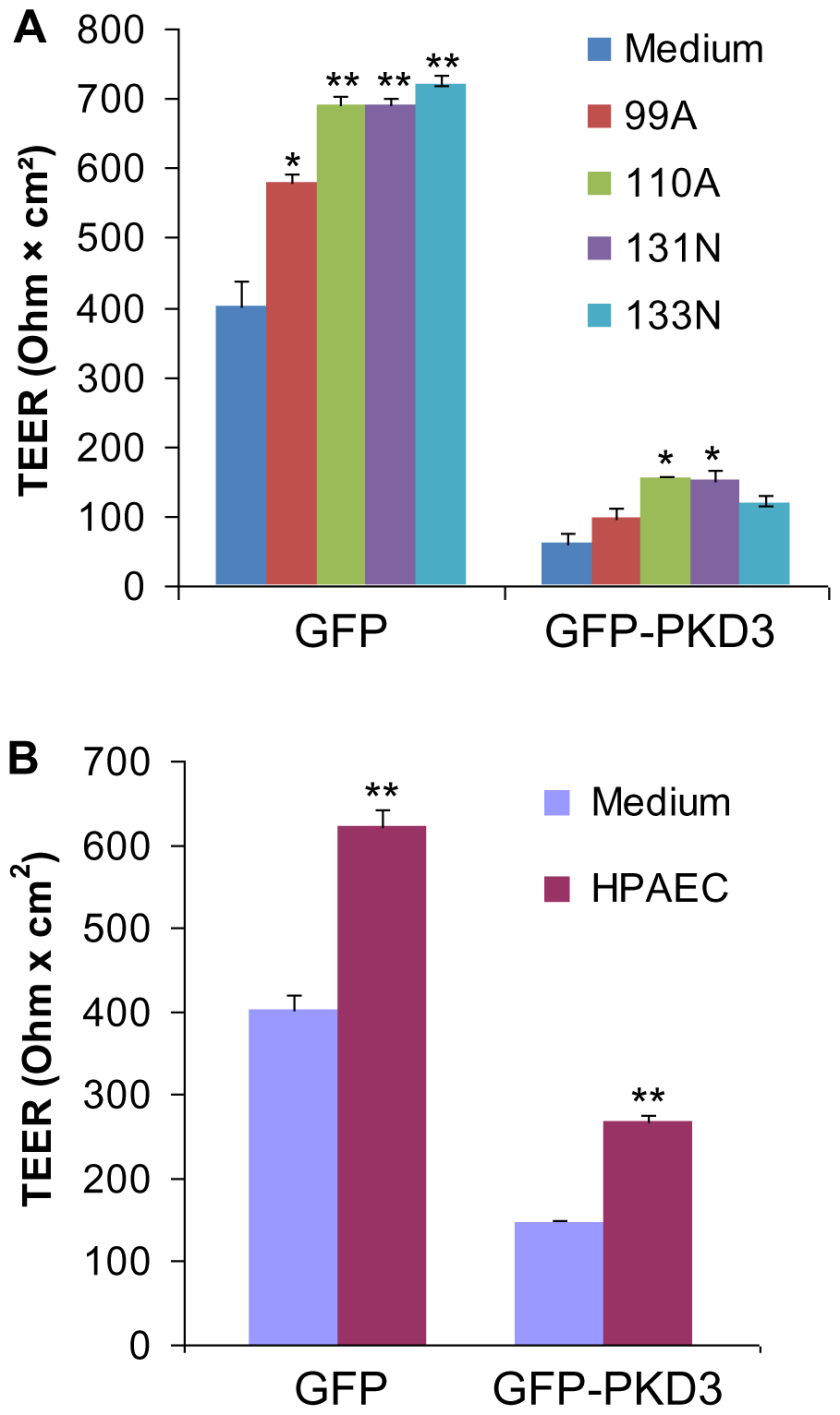
PKD promotes lung epithelial barrier permeability in the presence or absence of co-cultured primary lung fibroblasts or endothelial cells. A: pooled 16HBE14o- cells stably expressing control GFP or GFP-PKD3 as described [22] were grown on Transwell inserts in DMEM medium supplemented with 7.5% FBS in the presence or absence of co-cultured primary human lung fibroblasts derived from IPF lungs (99A and 110A) and normal subjects (131N and 13N) in the bottom chamber. TEER (Ohm×cm^2^) was measured on day 6 using an EVOMX voltohmmeter. B: pooled 16HBE14o- cells stably expressing control GFP or GFP-PKD3 were grown on Transwell inserts in EGM-2 medium with or without co-cultured HPAECs in the bottom chamber and TEER was measured on day 5. All data are means ± S.E. (n = 3). *, p<0.05; **, p<0.01 *versus* control GFP control cells. Similar results were obtained in three independent experiments.

Since PKD family kinases are increased and activated in IPF bronchiolar and alveolar epithelia, we next sought to assess the effect of PKD overexpression on lung epithelial cell biology. We have recently shown that overexpression of PKD family kinases disrupts the formation of apical intercellular junctions and their reassembly, impairs the development of TEER, and increases paracellular permeability to sodium fluorescein in 16HBE14o- human airway epithelial monolayers [22]. As lung epithelial cells interact with proximate fibroblasts and endothelial cells, we next assessed whether PKD could also promote lung epithelial barrier dysfunction in the presence of co-cultured primary lung fibroblasts or endothelial cells. TEER reflects the paracellular and transcellular resistance and is a sensitive measure of barrier integrity. We found that TEER of control 16HBE14o- cell monolayers on the Transwell inserts was significantly increased by co-culturing with primary lung fibroblasts derived from IPF lungs (99A and 110A) and normal subjects (131N and 13N) (Fig. 7A) or with HPAECs (Fig. 7B) in the bottom chamber. Moreover, 16HBE14o- cells overexpressing GFP-PKD3 developed a low TEER, and the TEER was increased but not reversed to the level of control GFP cells in the presence of co-cultured lung fibroblasts (110A and 131N) (Fig. 7A) or HPAECs (Fig. 7B). These findings indicate that lung fibroblasts and endothelial cells protect epithelial barrier integrity; however they can not reverse the defect in TEER development by PKD overexpression. Additionally, it appears that IPF and normal lung fibroblasts have a comparable ability to protect epithelial barrier function.

## Discussion

IPF, the most common form of the idiopathic interstitial pneumonias, is a chronic, relentlessly progressive and usually fatal lung disease of unknown etiology, and for which no effective treatments exist so far [1], [2], [3], [4]. Although the pathogenic mechanisms that underlie IPF are not clear, a growing body of evidence indicates that IPF is driven by abnormally activated AECs. It is believed that repetitive epithelial injury leads to aberrant activation of AECs. These AECs produce mediators to stimulate the proliferation of resident mesenchymal cells, to attract circulating fibrocytes, and to promote the epithelial to mesenchymal transition, resulting in the formation of fibroblast and myofibroblast foci. Activated myofibroblasts then secrete excessive amounts of extracellular matrix with the subsequent destruction of the normal lung architecture and the loss of alveolar spaces [2], [4], [32]. It has been shown that the activated AECs, such as hyperplastic type II pneumocytes and regenerative AECs, produce a number of chemokines, cytokines and growth factors, including TGFβ, PDGF, TNFα, and endothelin I [32], [33]. In this study, we found that PKD family kinases were increased and activated in the hyperplastic and regenerative AECs lining remodeled fibrotic alveolar septa and/or fibroblast foci in IPF lungs. In contrast, PKD family kinases were not apparently increased and activated in IPF fibroblasts or myofibroblasts compared with regenerative AECs. These findings indicate that expression levels of PKD family kinases differs in mesenchymal cells within the injured lung and suggest that the proportion of epithelial cells that have undergone mesenchymal transition likely lose expression of these kinases as part of the phenotypic change. While the differences between epithelial and mesenchymal cell expression of PKD family kinases were clear, the findings in isolated cells were not tested, representing a potential limitation of these analyses. It is possible, however, that the isolation procedures could affect expression of these kinases, so we relied on immunohistichemical analyses as our primary assessment.

We and others have reported that PKD family kinases are critical for cell proliferation, the production and secretion of cytokines and growth factors in numerous types of cells in response to various stimuli [12], [13], [14], [34], [35], [36], [37]. It is possible that activated PKD may contribute to the abnormal behavior of hyperplastic or regenerative AECs and the subsequent progressive course of IPF. Our studies support the hypothesis. We have recently shown that PKD overexpression or activation in lung epithelial cells markedly increased the cell barrier permeability by disrupting tight junctions [22]. Moreover, we found that lung fibroblasts and endothelial cells can protect epithelial barrier integrity; however they can not reverse the defect in epithelial barrier function caused by PKD overexpression. These findings suggest that the increased expression and activation of PKD in regenerative AECs may result in an impaired barrier integrity, which could facilitate the transit of profibrotic factors in IPF alveoli to reach mesenchymal cells that could proliferate with distortion of the alveolar compartment. Indeed, the epithelial barrier dysfunction or disruption is a critical factor in the pathogenesis of IPF and bleomycin-induced lung injury and fibrosis [38], [39].

We found that PKD family kinases were increased and activated in alveolar macrophages in almost all IPF subjects examined. It has been recently shown that PKD1 is essential for TLR ligand-induced macrophage activation and cytokine production [15] and that PKD inhibition suppresses microbial Ag-induced hypersensitivity pneumonitis in mice [16]. There is a current belief that chronic inflammation influences the pathogenesis of IPF. Recent studies have suggested that macrophage polarization from an M1 to M2 phenotype may promote fibrogenesis [40]. It is likely that PKD family kinases play roles in regulating alveolar macrophage activation and function in IPF, which merits future investigation.

Increasing evidence indicates that the disorders of small airways are involved in the pathogenesis of IPF [23], [24], [25]. Occlusion of the upstream airways may cause or promote the injury-induced damage to the downstream lung parenchyma. Fulmer et al reported that 94% of the IPF patients had peribronchiolar fibrosis, peribronchiolar inflammation or bronchiolitis, and suggested that IPF is a disease of small airway and alveoli [23]. Bronchiolar hyperplasia with extension to the pleural surface was also identified in 88% of IPF cases in a later study [24]. It has been recently reported that the expression of mucin 5B is increased in IPF distal airways and honeycomb cysts [25]. Interestingly, we found that PKD family kinases were highly increased and activated in all the IPF bronchiolar epithelia, including honeycomb cysts. Specifically, PKD1 was abundantly expressed and activated in cilia of BECs, and PKD2 and PKD3 were expressed in the cytoplasm and nuclei of IPF BECs. PKD1 has been shown to be a negative regulator of actin cytoskeleton [8]. It would be interesting to know whether PKD1 negatively regulates the motility of tubulin-containing cilia, the production of mucins, and the subsequent mucus clearance function of airways in IPF.

We also found that LPA, thrombin, and poly-L-arginine strongly activated PKD in both primary small airway epithelial and A549 alveolar cells. LPA and thrombin are profibrotic factors and are implicated in the pathogenesis of pulmonary fibrosis [29], [30]. In particular, LPA levels in bronchoalveolar lavage fluids are significantly increased in IPF patients; and knockout of LPA receptor-1 markedly suppresses the bleomycin-induced pulmonary fibrosis in mice [30]. Poly-L-arginine is a highly charged cationic polypeptide that is similar in structure and function to the active moiety of major basic protein secreted from eosinophils [31]. In contrast, some well-known profibrotic factors, such as TGFβ and PDGFβ essentially did not affect PKD activation in the epithelial cells. These data suggest that PKD family kinases are not the effectors of these fibrogenic factors but rather may regulate the expression and secretion of these factors from activated AECs and/or macrophages in IPF lungs.

In summary, we have obtained substantive evidence indicating that PKD family kinases are increased and activated in IPF bronchiolar and alveolar epithelia as well as lung macrophages. PKD is predominantly activated by LPA, thrombin and poly-L-arginine in lung epithelial cells and promotes lung epithelial barrier dysfunction and permeability. PKD family kinases may represent a potential target for the development of novel and efficacious therapeutic intervention in IPF.
